# Co-production in public health research grant writing: engaging underserved migrant mothers in the UK

**DOI:** 10.1093/eurpub/ckac131.515

**Published:** 2022-10-25

**Authors:** K Stevenson, K Ogunlana, Y Ciftici, M Knight, R Aldridge, F Stevenson

**Affiliations:** 1 Faculty of Public Health and Policy, LSHTM, London, UK; 2 Institute for Health Informatics, University College London, London, UK; 3 Doctors of the World UK, London, UK; 4 National Perinatal Epidemiology Unit, University of Oxford, Oxford, UK; 5 Primary Care and Population Health, University College London, London, UK

## Abstract

**Background:**

In the UK, one in three births is to a non-UK born woman, but there is a gap in co-produced research to explore their experiences. The National Institute for Health and Care Research (NIHR) defines co-production as ‘an approach in which researchers, practitioners, and members of the public work together, sharing power and responsibility'. This project co-produced a grant proposal to improve maternity care for underserved migrant women in the UK. We reflect on transferrable learning for engaging those whose voices are less often heard in grant writing.

**Methods:**

An expert by experience, an underserved migrant woman who gave birth in the UK, joined the research team. Four online engagement workshops were conducted; two involved only migrant women, two were multi-disciplinary. 26 underserved migrant women attended. NIHR INVOLVE public involvement guidance was consulted.

**Results:**

Women said they were often asked about negative experiences which felt disempowering, and rarely asked about solutions. Thus, we shifted the focus of our work to co-designing solutions. Women said that having an expert by experience co-host workshops encouraged engagement, so we integrated this into our methods. Some women were uncomfortable in professional groups. Thus, our proposed steering and focus groups will have an expert by experience subgroup with elected members attending multi-disciplinary groups. We will engage mostly online as women preferred this to enable flexibility with childcare. The lead expert by experience helped form the proposal through brainstorming, co-drafting, and feedback; experts by experience commented on the draft via email and workshops. The lead expert by experience wished to gain further experience of research methods, for which we requested additional funding.

**Conclusions:**

This project highlights the immense potential for co-production in public health research, and the value of adapting research planning to maximise the voices of the less often heard.

**Key messages:**

• Engaging experts by experiences in public health research planning is key to ensuring our work addresses the needs of underserved communities.

• Co-Production of research requires determination to involve those whose voices are less often heard from the beginning of the research process, and to commit to joined working throughout.

